# Synthesis of Ti-Al-xNb Ternary Alloys via Laser-Engineered Net Shaping for Biomedical Application: Densification, Electrochemical and Mechanical Properties Studies

**DOI:** 10.3390/ma15020544

**Published:** 2022-01-12

**Authors:** Lehlogonolo Rudolf Kanyane, Abimbola Patricia Idowu Popoola, Sisa Pityana, Monnamme Tlotleng

**Affiliations:** 1 Department of Chemical, Metallurgical and Materials Engineering, Tshwane University of Technology, P.M.B. X680, Pretoria 0001, South Africa; popoolaapi@tut.ac.za; 2 Laser Materials Processing Group, National Laser Center CSIR, Pretoria 0001, South Africa; SPityana@csir.co.za (S.P.); MTlotleng@csir.co.za (M.T.); 3 Department of Mechanical Engineering Science, University of Johannesburg, Auckland Park Campus, Johannesburg 2012, South Africa

**Keywords:** lens, microhardness, densification, anti-corrosion

## Abstract

The lives of many people around the world are impaired and shortened mostly by cardiovascular diseases (CVD). Despite the fact that medical interventions and surgical heart transplants may improve the lives of patients suffering from cardiovascular disease, the cost of treatments and securing a perfect donor are aspects that compel patients to consider cheaper and less invasive therapies. The use of synthetic biomaterials such as titanium-based implants are an alternative for cardiac repair and regeneration. In this work, an in situ development of Ti-Al-xNb alloys were synthesized via laser additive manufacturing for biomedical application. The effect of Nb composition on Ti-Al was investigated. The microstructural evolution was characterized using a scanning electron microscope (SEM) equipped with energy dispersive spectroscopy (EDS). A potentiodynamic polarization technique was utilized to investigate the corrosion behavior of TiAl-Nb in 3.5% NaCl. The microhardness and corrosion behaviour of the synthesized Ti-Al-Nb alloys were found to be dependent on laser-processing parameters. The microhardness performance of the samples increased with an increase in the Nb feed rate to the Ti-Al alloy system. Maximum microhardness of 699.8 HVN was evident at 0.061 g/min while at 0.041 g/min the microhardness was 515.8 HVN at Nb gas carrier of 1L/min, respectively.

## 1. Introduction

Titanium and its alloys, along with stainless steel (SS) and Co-Cr alloys, are the most used metallic biomaterials in cardiovascular application [1]. This material was adopted following clinical trials carried out in the late 1930s that provided evidence that Ti exhibited similar biological characteristics to SS and cobalt alloys. Titanium became a prospective material because of its interesting features, such as of lightness and equivalent strength when compared to both SS and cobalt alloys [1,2].

Metallic biomaterials such as titanium and its alloys are presently used as structural materials in artificial hip joints, artificial dental roots, bone plates and screws. These classes of materials are primarily utilized in implants that replace hard tissue [3,4,5]. Among biometals, titanium alloys have a high specific strength, biocompatibility and good corrosion resistance, and they display the most appropriate physical characteristics for biomedical applications [6,7,8]. On the other hand, titanium and titanium alloys cannot meet all the desired clinical requirements. The most frequently used titanium alloy for medical applications today is Ti6Al4V [9]. Nevertheless, Ti6Al4V alloys are normally restricted to non-friction occasions due to the low hardness and poor wear resistance properties and they also present poor corrosion resistance properties in aggressive environments [10,11]. Cruz, Souza [12] stated that the degradation of titanium implants is as a result of a combination of micro-motions, the working environment and poor wear resistance.

In recent research, hydroxyapatite (HAp) is considered the most biocompatible material for use in the replacement and regeneration of bone material [13]. The main component that is present in the hard-body tissues is a hydroxyapatite nanostructure [11]. HAp is widely utilized as a bioactive coating material in metallic implants in biomedical applications due to their outstanding biocompatibility, osteo-conductivity and its chemical and structural similarity to natural bone. HAp is also coupled with quicker implant fixation along with a strong bond between implants and the living bone. However, the way the state of the art nano-technology can be exploited to fabricate hydroxyapatite nanophase with similar properties compared to natural hydroxyapatite found in hard tissues presents many challenges [13,14,15,16].

Hence, the demand for new materials with enhanced properties for biomedical applications is growing. In the coming decades the mortality and morbidity rates of CVD disease are anticipated to rise. Unfortunately, the expected rise in these rates brings along hefty financial burdens [17]. Hence, new technologies for fabrication of new biometals need to be implemented. The high strength-to-weight ratio property of titanium aluminide (TiAl)-based intermetallic alloys makes researchers view this type of material as a potential biometal. Due to the lack of ductility in TiAl alloys, additional alloying elements to improve the ductility of the alloy are investigated for potential biometals.

Many studies have been conducted to investigate titanium-based alloys’ improvements in oseointegration with bones, which lead to poor bonding between bone tissue and implants [18,19,20,21,22,23]. Laser Engineered Net-shaping (LENS) in Additive Manufacturing (AM), is a breaking-edge manufacturing technique presenting the possibility of changing the perception of design and manufacturing as a whole. LENS manufacturing provides an opportunity to produce Ti alloys with layer by layer melting and the potential to design complex components for biomedical applications. The technique is also recognized for reducing the necessary time and machining-cost in such processes [19,22,24]. Many authors have used LENS to develop titanium alloys for biomedical application [25,26]. The scope of industrial application of LENS technology is largely reliant on process efficiency, precision of design-to-build, build consistency and repeatability, all of which depend on a developed knowledge of the process parameters and powder characteristics [27,28]. Das, Balla [24] fabricated cp-Ti using LENS for potential biomedical implants application. The author also studied the influence of laser processing parameters and revealed that the density of the cp-Ti decreases with an increase in the powder-feed rate and that laser power decreases as a result of partially molten powder which creates porosity. Branzoi, Iordoc [29] studied the surface characteristics of Ti-based (TiAl6V4, Ti6Al7Nb and TiNi) alloys in a fetal bovine serum solution. The results indicated that the TiAlNb alloy had an outstanding corrosion behaviour compared to the TiAlV alloy, and that Nb was more stable and less toxic compared to V.

The literature shows that laser additive manufacturing can be used to fabricate Ti-Al based alloys. Information on the fabrication of TiAl based alloys fabricated via in situ laser additive manufacturing for biomedical applications is limited. In this research, attempts were made to produce defect-free Ti-Al-xNb-based 3D printed samples, with improved mechanical properties, high densification and good electrochemical behaviour using direct laser metal deposition for potential biomedical application.

## 2. Materials and Methods

### 2.1. Materials

Pure elemental powders of Ti and Al were used as feedstocks in this study. Ti and Al powders were sphere-shaped, with a particle distribution size range of 45–90 µm. The powders were supplied by TLS Technik (Bitterfeld-Wolfen, German) while Nb (irregular in shape) was supplied by Weartech (Johannesburg, South Africa). Powders were deposited onto Ti6Al4V substrates with dimensions of 140 × 140 × 7 mm^3^.

### 2.2. Methods

#### 2.2.1. Laser Metal Deposition

LENS additive manufacturing is a process used for fabricating high quality components for advanced biomedical and engineering purposes. The CSIR’s LENS^®^ 850-R system, manufactured by Optomec, Albuquerque, NM, USA), was utilized to synthesize Ti-Al-xNb alloys on a grade 5 titanium alloy (Ti-6Al-4V) substrate. This LENS system uses a 1 KW IPG fiber laser mounted onto the deposition head so that the head and laser beam could be manipulated or controlled simultaneously. The system was received as standard, with two hoppers used for binary laser in situ alloying or FGMs research. For research regarding laser in situ alloy ternary and powders, GTV (Verschleiss-Schutz Gmbh, Luckenbach, Germany) powder hoppers are externally attached to the LENS to produce 3 modified hoppers in the LENS set-up. All powder feeders are connected to the bulk Argon gas supply that is used as a carrier gas during processing. The modified LENS set-up was then automatically controlled from a central computer working station that was installed an Optomec software, version 3.1.6. Table 1 reports the process parameters that were used to manufacture the Ti-Al-xNb coupons studied in this paper.

The Ti gas carrier was set to 4.2 L/min, while Al was 2.4 L/min which equated to 2.21 g/min and 0.48 g/min, respectively. The LENS set-up was composed of three hopers of which Ti, Al and Nb powders were carried by the Argon gas to the deposition zone. The development of Ti-Al-Nb occurred on the base plate that was continuously heated around the temperature below the phase transformation in relation to the Ti-Al phase diagram. Table 2 presents the sample code of the manufactured Ti-Al alloys used to fabricate the Ti-Al alloy at an Nb gas carrier flowrate of 1 L/min and the equivalent Nb powder feed rate in g/min.

#### 2.2.2. Sample Preparation and Analyses

The synthesized cube samples were cut from the substrate and mounted in an epoxy- based resin, grinded and polished using typical metallography procedures and etched with a Kroll agent solution to evaluate the microstructural evolution. Olympus BX51M was mounted on a SC30 camera, and was used for microstructural visualisation while Joel, JSM-6010Plus/LAM scanning electron microscopy (SEM) (Peabody, MA, USA) equipped with energy dispersive X-ray (EDS) and used for microstructural and chemical composition analyses. The phase of the alloys was identified using the X’Pert PANalytical X-ray diffraction machine (PANalytical Empyrean model, Malvern Panalytical Ltd., Royston, UK) that uses a Cu source. During data acquisition, the Cu source was excited with a current and voltage of 40 mA and 54 kV, respectively.

#### 2.2.3. Microhardness

The Vickers microhardness (HVN) measurements of the synthesized alloys were analyzed using an Zwick/Roell Indentec (ZHVµ) microhardness tester machine (Zwick Roell AG, Ulm, Germany). An indention load of 500 kgf was applied on the surface of the samples with a dwell time of 15 s. The fabricated sample surfaces were indented randomly at ten different positions and the mean value was recorded.

#### 2.2.4. Density 

Archimedes’ principle was employed to measure the densities of the fabricated samples using a Density-o-meter measuring scale. For accurate statistical data, five measurements were carried out on each of the samples and the relative density was calculated as a function of the theoretical and measured density of the developed Ti-Al-Nb.

#### 2.2.5. Corrosion Test

Corrosion testing was performed using the autolab pontetionstat to determine the linear polarisation of the fabricated samples. The samples were tested in an NaCl environment as one of the main constituents of human body fluid and the tests were performed at a scan rate of 0.01, with a start and stop potential of −1.5 V and 1.5 V, respectively.

## 3. Results and Discussion

### 3.1. Microstructural Results

**Figure 1:**
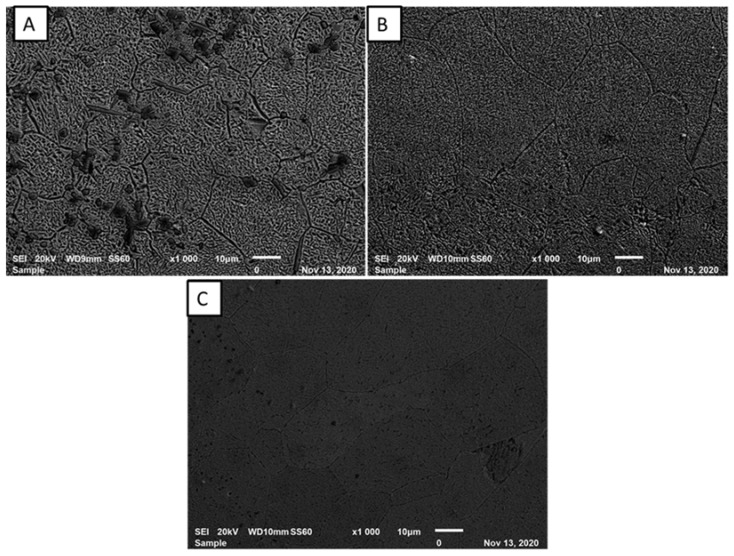
SEM images of Ti-Al-xNb synthesized at different Nb feed rate (**A**) A; (**B**) A1; (**C**) A3 and 1 L/min Nb gas carrier.

**Figure 2:**
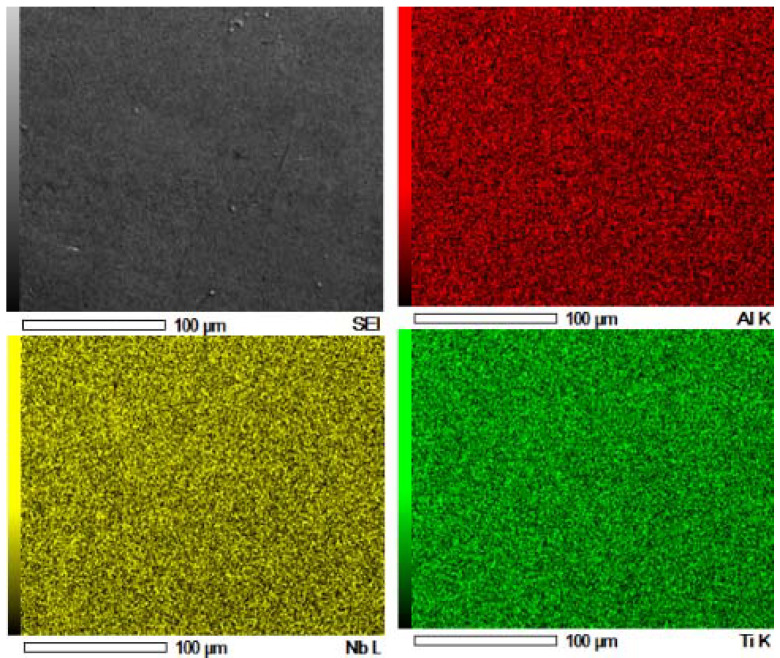
EDS map of synthesized Ti-Al-xNb (A1) at 0.052 g/min Nb feed rate.

Figure 1 present the microstructural evolution of laser synthesized Ti-Al-Nb alloy at 26 in/min and laser power of 450 W. The effect of Nb feed rate on TiAl matrix was studied from 0.041 to 0.061 g/min. The difference in the microstructural evolution of the laser fabricated alloy occurred due to the variation of the Nb feed rate which resulted in a unique compositional effect hence, leading to unique micrographs. The developed alloys resulted in non-homogenous structures with irregular Ti rich precipitates. The fabricated Ti-Al-Nb alloys were free from pores and cracks or any initiation of stress. We also observed that, with an increase in Nb rpm, the alloy grains became finer. According to Malatji, Popoola [30], Nb has the capability to induce grain refinement in the alloy matrix and can also form a solid solution with other incorporated elements. At 0.041 g/min Nb feed rate (5at% Nb), it is clear that the grains are large in size as compared to samples developed at 0.052 g/min (A1) and 0.061 g/min (A3). The nature of laser manufacturing could also play a role in the observed general fine microstructure, as a result of fast cooling of the melt pool affording less time for grain growth [31]. The benefit of Nb incorporation in titanium was reported to stabilize the formation of β-phase (bcc) with high strength but moderate ductility. The addition of Nb in a Ti-Al alloy matrix can act as BCC β-stabilizer in both γ-TiAl and α_2_-Ti_3_Al phases, which leads to the formation of ordered orthorhombic Ti_2_AlNb phases. Moreover, Nb acts as a substitutional or interstitial solute in the crystal lattice matrix [32]. This helps to induce the distortion of the crystal lattice, resulting in a reduction of dislocation movements, which lead to an improvement in the mechanical properties of the alloys [33]. Figure 2 presents images of EDS elemental mapping of the alloy at a given area, where the elemental maps show a uniform distribution of particles for Nb, Ti, and Al. The images also indicate that titanium is the richest phase in the alloy by atomic percentage.

**Figure 3:**
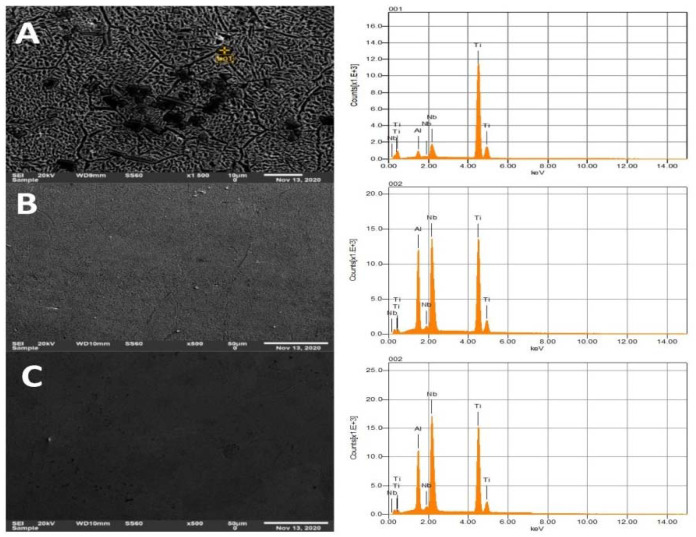
EDS images of synthesized Ti-Al-xNb; (**A**) A, (**B**) A1 and (**C**) A3.

Figure 3 presents SEM images of Ti-Al-xNb alloys with varied Nb feed rates along with the EDS analysis graphs of the respective alloys. Figure 3A has a distinct dark spots phase which is an indication of the partial melted titanium rich particles as suggested by the EDS, and this affects the special distribution of titanium particles within the alloy. From the elemental composition of the developed alloys (Table 3), it can also be noticed that as the Nb feed rate increased, the amount of Nb in the matrix of the alloys also increased, which can also be observed on the Nb peaks apparent in Figure 3A–C. Table 3 also present the theoretical and the actual density of the in situ laser fabricated Ti-Al-Nb alloys. The densities were measured by Archimedes’ principle.

### 3.2. Phase Analysis Results

**Figure 4:**
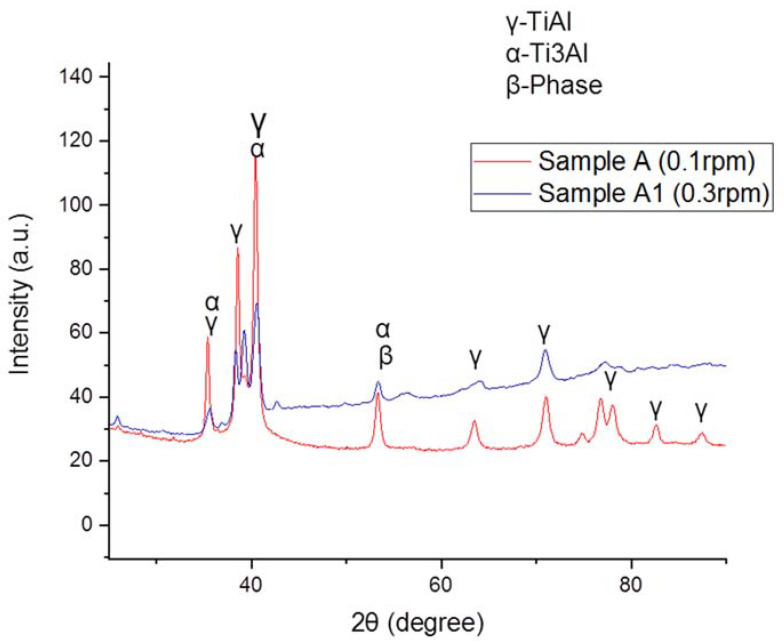
XRD plots of Ti-Al-Nb alloys.

Figure 4 represent the XRD pattern of the 3D printed Ti-Al-xNb via laser in situ alloying, fabricated at different laser processing parameters. The sample A presented major diffraction peaks of 38.21°, 40.45°, 42.62°, 56.39°, 70.74°, 78.75°, 83.01° and 88.03° their inter-planar distance of 2.35 Å, 2.23 Å, 2.12 Å, 1.63 Å, 2.13 Å, 1.71 Å, 1.67 Å and 1.33 Å, respectively. At 14at% Nb (A1) content, the diffraction peaks were found at 34.86°, 39.89°, 42.48°, 52.78°, 56.06° and 70.41° and inter-planar distances of 2.57 Å, 2.25 Å, 2.13 Å, 1.73 Å, 1.64 Å and 1.33 Å were found, respectively. The XRD spectrum of sample A (5at% Nb) shows the major phases (α-phase and γ-phase) present and the high peaks. The same peak was slightly reduced at sample A1 (10at%). Normally, the shift in the crystals structure and peak intensity decrease indicate atomic rearrangement. Furthermore, peak broadening, appearance and disappearance can be noticed with an increase in Nb content. A disappearance of γ phase can be noticed at 78.75°, 83.01° and 88.03° (2θ). The peak intensity decreased due to the dissolution of unstable phases to form stable phases with increasing Nb content. This is an example of twinning in Ti-Al-xNb. The synthesized alloys presented a higher fraction of γ phase in both powder-feed rates. From the study of [34], the combination of the β and γ phases are known to have moderate ductility and to induce toughening, whereas the α_2_ phase imparts strengthening in the advanced γ-TiAl alloys which makes it suitable for potential biomedical application. The present phases can be related to SEM images, as presented β and γ phases (Figure 1).

### 3.3. Densification Results

**Figure 5:**
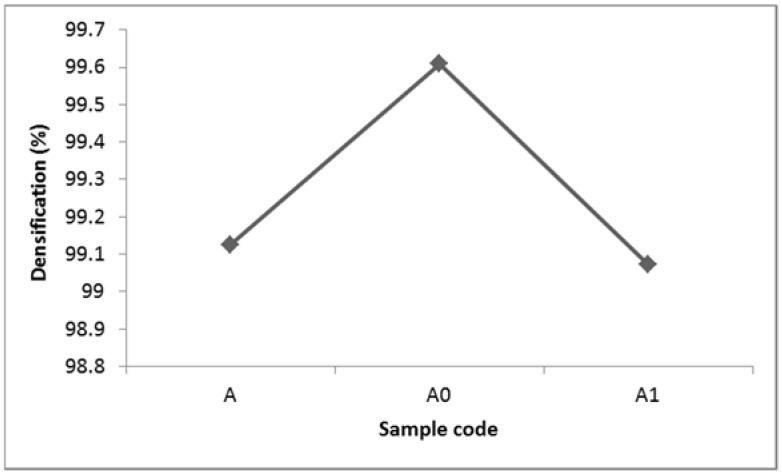
Densification results of synthesized Ti-Al-xNb.

Figure 5 present the effect on Nb on the densification properties of Ti-Al-xNb developed via laser in situ alloying. The principle used by Archimedes’ states the difference in weight of the material measured in air along with the sample material measured in suspended water as the volume of an object. The Archimedes’ equation of relative density is presented Equation (1);

(1) $$r_{s}=\frac{\left(m_{s}\cdot r_{w}\right)}{\left(m_{s}-m_{w}\right)}$$

The Ti-Al-xNb alloys densities (r_s_) in g/cm^3^ are derived from the amount of the product of the mass of the alloy in air (m_s_) in grams (g) and the density of water at room temperature (r_w_) in g/cm^3^ with the difference between the mass of the alloy in air and the mass of alloy in water (m_w_) in g. Generally, the developed alloys are much denser with a densification of more than 99%. This can be attributed to the high laser power (450 W) used to melt the Ti, Al and Nb powders, which resulted in minimal micro-pores present in the fabricated alloys. Furthermore, it is known that high temperatures during laser fabrication result in high particle-to-particle fusion, which result in a good relative density of the material [35]. Authors have mentioned the relationship between the density of the material and its microhardness properties. According to [36], the densification of alloys has a significant influence on its microhardness properties. High alloy densification has been associated with improved microhardness value. According to [37], high densification properties of titanium aluminides can also be attributed to particle rearrangement and the homogeneous distribution of phases which are more favored for biomedical applications. 

### 3.4. Microhardness Results

**Figure 6:**
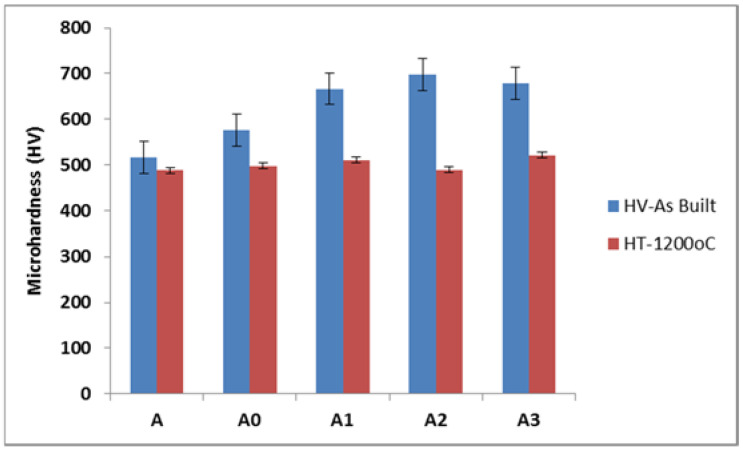
HT effect on microhardness properties of developed Ti-Al-Nb.

Figure 6 depicts microhardness properties of the synthesized Ti-Al-xNb at different Nb feed rates. The results present the mean microhardness value for Ti-Al-xNb with varying compositions as a result of the different feed rate of Nb. The samples were indented randomly at 10 different points and the average value was presented. Vickers microhardness tests were performed to assess the tendency of the developed alloys to resist plastic deformation. It is obvious from the presented results that the Ti-Al-xNb at the Nb gas carrier of 1 L/min and feed rate of 0.055 g/min led to better enhancement compared to other fabricated alloys, which presented the maximum microhardness value of 699.8 HVN. However, as the Nb flow rate increased to 0.061 g/min (A3), the microhardness slightly reduced to 684.6 HVN. There was a clear relationship between the Nb feed rate and microhardness properties at 1 L/min gas carrier. It can be confirmed from the graph that the increase in the microhardness was a result of the Nb feed rate increase. Generally, the strengthening mechanism of the developed Ti-Al-xNb can be attributed to the rapid cooling of the melt pool during laser manufacturing, which results in small grains, known for their good mechanical properties. According to Masina, Skhosane [38] and Malatji, Popoola [30], laser processing parameters also play an important role in improving microhardness properties of laser-developed materials. The incorporation of Nb as an alloying element is also known for improving mechanical properties such as high microhardness, high fracture toughness and outstanding wear resistance, which makes it applicable for biomedical purposes [3,39,40].

The annealing heat treatment effect on the microhardness properties of synthesized Ti-Al-xNb alloys was investigated. The samples were heat treated at a temperature of 1200 °C for 2 h in a muffle furnace. LENS-developed samples are known for low ductility. The reason relates to the fact that laser material processing results in rapid cooling that takes place in the process. Annealing heat-treatment helps with stress relief and re-crystallization in the alloys. The dramatic microhardness decrease can be attributed to the fact that annealing could result in rapid grain growth as the samples are cooled slowly in the furnace [41,42,43].

### 3.5. Corrosion Results

**Figure 7:**
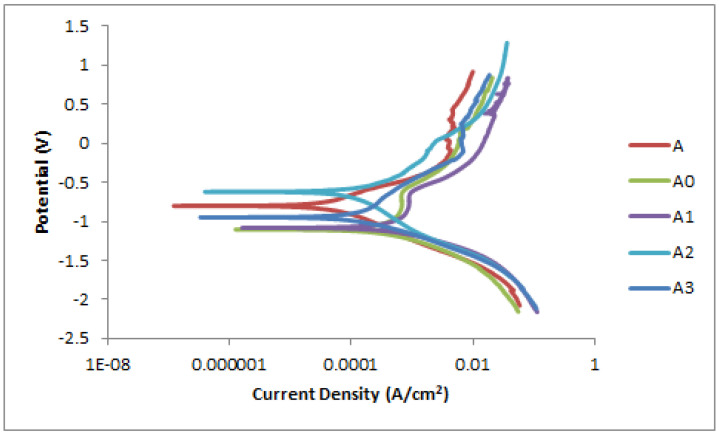
Linear polarization curve of Ti-Al-xNb at 1 L/min Nb gas carrier in NaCl.

Figure 7 represents the potentiodynamic polarization of LENS manufactured Ti-Al-xNb alloys at various Nb feed rates and a gas carrier of 1 L/min. The native film (oxide layer), which forms spontaneously at room temperature on the Ti alloys’ surface provides them with an outstanding biocompatibility property. In most cases, biocompatibility of biomaterial implants is measured by the response of host post implantation. The human body is considered to be a very hostile environment for any foreign objects, including biomaterials. What makes the human environment aggressive is its components, such as blood and other body fluids such as water, proteins, plasma and amino acids. These constituents are composed of various ions such as chloride, phosphate, and bicarbonate, sodium, potassium, calcium, magnesium ions, etc. [44]. Among these ions, chloride ion is recognized as the most aggressive and corrosive to metals. As the human body contains many salts, the electrochemical behavior of the Ti-Al-xNb biomaterial was examined in 3.5% NaCl medium to understand the response of Ti-Al based alloy in Cl conditions for potential biomedical applications. The plots show a shift in the pitting potential of the alloys, along with a trend of passivating behavior. There was a positive shift of corrosion potential (E_corr_) of the synthesized samples from sample A (0.041 g/min) to A3 (0.052 g/min). According to Krzywicka, Antoszewski [45], in the case of more positive E_corr_ values, the sample shows a greater ability to develop a protective oxide layer to inhibit corrosion. The high potential of −0.62267 V was evident in sample A2 with a current density of 8.28 × 10^−5^ A/cm^2^ as indicated in Table 4 of the corrosion-dynamic fit of the Ti-Al-based samples. We observe no clear relationship between the Nb feed rate and corrosion-resistance behaviour. Additionally, a larger active range on the anodic branch is observed with sample A, A2 and A3 when compared to samples A0 and A1 which show oxide-protective behaviour, further indicating a greater susceptibility to corrosion after passivation is attained.

#### SEM Images of Corroded Samples in 3.65% NaCl Solution

**Figure 8:**
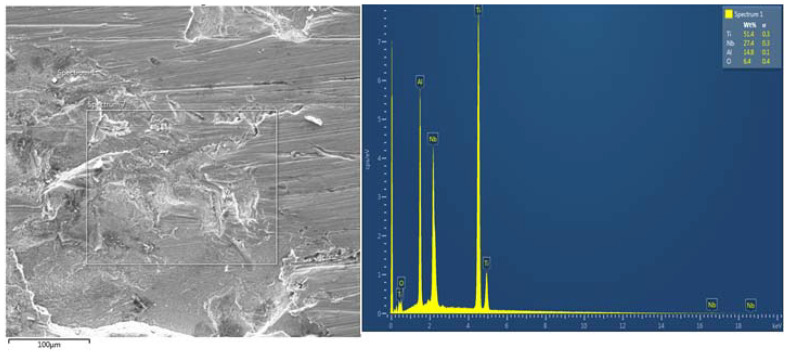
SEM/EDS of corroded sample in 3.65% NaCl.

Figure 8 presents the SEM/EDS microstructure of corroded Ti-Al-xNb samples developed both at A1 and a gas carrier of 1 L/min. The morphological evolution of the fabricated Ti-Al-xNb samples demonstrates the existence of the oxide film of the elements on the alloy surface. From the EDS analysis of corroded Ti-Al-xNb samples, it can be observed that the samples presented a depleted Nb and Al content, respectively. The presence of a high oxygen content shows that the formation of oxide products occurs on the surface of the alloys. These products result in cation vacancy diffusivity across the oxide layer and consequently cause more defect sites and a breakdown of the passive film oxide. In support of the TiO_2_ breakdown mechanism, the localized exposure of TiO_2_ to chloride ions encourages the formation of unstable titanium oxychloride [46]. The study conducted by Shi et al. [47] showed that the presence of Ti and Nb in s 3.5 wt.% NaCl solution presented outstanding anticorrosion behaviour, which is attributable to the fact that Ti and Nb content facilitate the formation of the protective oxide layer (TiO_2_ and Nb_2_O_5_) on the surface of Ti-Al-xNb alloys. It is evident from literature that the Al_2_O_3_ layer is porous and because of this, it allows for the penetration of detrimental Cl^−^ ions on the exposed surface [48,49,50]. Moreover, the presence of the Cl elemental peak on the EDS could be a result of corrosion products containing Cl. These products may include AlCl_3_, TICl_4_ as identified through Equations (2)–(4). The following reactions may occur during corrosion tests:

Al + 3NaCl → AlCl_3_ + 3Na (2)

Ti + 4NaCl → TiCl_4_ + 4Na (3)

Nb + 5NaCl → NbCl_5_ + 5Na (4)

## 4. Conclusions

✓ Ti-Al-xNb was successfully synthesized as a biomaterial by means of LENS-additive manufacturing and the following conclusions were drawn:
✓ The EDS of the samples suggested that all incorporated elemental powders were available in the fabricated Ti-Al-xNb alloy.
✓ The effect of the Nb feed rate was sufficient to create different microstructural evolutions, as presented by SEM images
✓ Heat-treatment of the developed samples presented a significant decrease in microhardness with a maximum hardness value of 521.4 HV, which suggest that stress relief was achieved.
✓ A high potential of −0.62267 V was evident for sample A2, with a current density of 8.28 × 10^−5^ A/cm^2^.
✓ SEM/EDS of corroded samples presented no evidence of rust, however, oxide-protective layers are evident on the surface of the alloys

## Figures and Tables

**Table 1:** DED process parameters.

Parameter	Symbol	Set-Value	Unit
**Laser power**	P	450	W
**Laser spot size**	D	1.4	mm
**Deposition speed**	S	26	in/min
**Al powder**	M-Al	2.4	L/min
**Ti powder**	M-Ti	4.2	L/min
**Nb powder**	M-Nb	1.0	L/min

**Table 2:** Sample code and the Nb feed rate applied in developing Ti-Al based alloys.

Nb Feed Rate (g/min)	
**Sample Code**	
**A**	0.041
**A0**	0.043
**A1**	0.052
**A2**	0.055
**A3**	0.061

**Table 3:** Elemental composition (at%) and actual density of the developed alloys.

	Elements in Atomic %			Theoretical Density (g/cm^3^)	Actual Density (g/cm^3^)
**Sample**	Ti	Al	Nb		
**A**	56.22 (56)	38.75 (39)	5.04 (5)	4.06	4.0245
**A1**	47.34 (47)	42.44 (42)	10.22 (10)	4.22	4.2035
**A3**	51.34 (51)	34.89 (35)	13.77 (14)	4.50	4.4585

**Table 4:** Tafel results of Ti-Al-xNb LENS manufactured alloys in NaCl.

Sample Code	Ecorr, Obs (V)	Jcorr (A/cm²)	Corrosion Rate (mm/Year)	Polarization Resistance (Ω)
**A**	−0.80245	6.99 × 10^−5^	0.021175	1258.5
**A0**	−1.1062	3.63 × 10^−5^	0.082186	1140.83
**A1**	−1.0843	0.000267	0.081057	1128.75
**A2**	−0.62267	8.28 × 10^−5^	0.01023	2536.41
**A3**	−0.94834	0.000181	0.06053	1464.46
